# Potential Hormone Mechanisms of Bariatric Surgery

**DOI:** 10.1007/s13679-017-0276-5

**Published:** 2017-08-05

**Authors:** Georgios K. Dimitriadis, Manpal S. Randeva, Alexander D. Miras

**Affiliations:** 1 Division of Translational and Experimental Medicine, Clinical Sciences Research Laboratories, University of Warwick Medical School, Coventry, CV2 2DX UK; 2 Academic Division of Diabetes, Endocrinology and Metabolism, Imperial College London, Hammersmith Campus, London, W12 0NN UK; 3 Division of Translational and Experimental Medicine-Metabolic and Vascular Health, Warwick Medical School, University of Warwick, Coventry, CV4 7AL UK

**Keywords:** Bariatric surgery, Gastric banding, Sleeve gastrectomy, Roux-en-Y gastric bypass, Gut hormones, Gastrin, GLP-1, GLP-2, PYY, Ghrelin, CCK, GIP, Oxyntomodulin, Secretin, VIP, PP, Insulin, Glucagon, Somatostatin, Obestatin, Gustducin, FGF19, FGF21

## Abstract

**Purpose of Review:**

In recent years, the role of the gastrointestinal (GI) tract in energy homeostasis through modulation of the digestion and absorption of carbohydrates and the production of incretin hormones is well recognized.

**Recent Findings:**

Bariatric surgery for obesity has been a very effective method in substantially improving weight, and numerous studies have focused on intestinal adaptation after bariatric procedures. A number of structural and functional changes in the GI tract have been reported postsurgery, which could be responsible for the altered hormonal responses. Furthermore, the change in food absorption rate and the intestinal regions exposed to carbohydrates may affect blood glucose response.

**Summary:**

This review hopes to give new insights into the direct role of gut hormones, by summarising the metabolic effects of bariatric surgery.

## Introduction

Obesity, defined as a body mass index ≥30 kg/m^2^, has become a worldwide epidemic, considered among the greatest public health challenges of our time. An estimated global progression of obesity over the next decades indicates that by 2030, more than 1 billion adults will be obese [1]. Obesity can contribute towards multiple cardiometabolic co-morbidities, with debilitating consequences. The weight reductive effects of bariatric surgery have been well documented over the past decades, and bariatric surgery remains currently the most effective weight loss method mainly by GI tract volume restriction and/or beneficial metabolic sequelae [2]. The mechanisms underlying the metabolic effects of bariatric surgery remain elusive, but they are likely to be secondary to changes in the secretion of gut hormones and the transformation of the gastrointestinal lining [3]. This review article aims at elucidating the potential hormone mechanisms of the most commonly used bariatric procedures (Table 1).

**Table 1:**
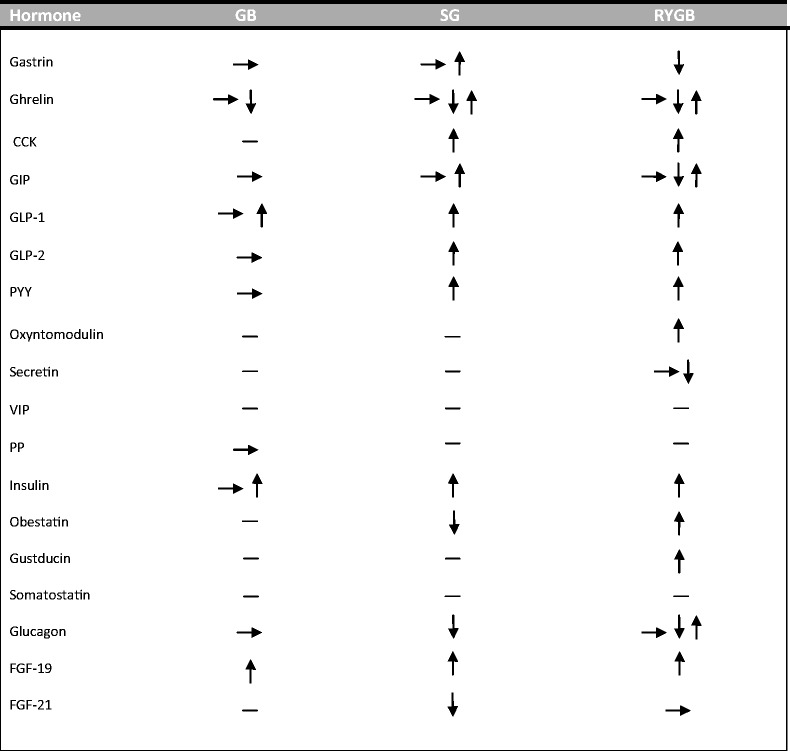
The effects of the most common bariatric procedures on gut hormone regulation

More than one arrow means that data are conflicting

*GB* gastric banding, *SG* sleeve gastrectomy, *RYGB* Roux-en-Y gastric bypass, *CCK* cholecystokinin, *GIP* glucose-dependent insulinotropic polypeptide, *GLP-1* glucagon-like peptide 1, *GLP-2* glucagon-like peptide 2, *PYY* polypeptide YY, *VIP* vasoactive intestinal polypeptide, *PP* pancreatic polypeptide, *FGF19* fibroblast growth factor 19, *FGF21* fibroblast growth factor 21, *→* no change, *↓* decreased, *↑* increased, *–* unknown

## Procedures

Bariatric procedures, such as Roux-en-Y gastric bypass and vertical sleeve gastrectomy, cause substantial and durable weight loss in both humans and rodents. Lately, these surgical interventions have also been termed metabolic due to the substantive metabolic changes beyond body weight loss alone. The most popular interventions at present are gastric banding, sleeve gastrectomy and the Roux-en-Y gastric bypass.

### Gastric Banding

Gastric banding (GB) includes the placement of a silicone ring around the stomach to create a small upper gastric pouch at the bottom of the oesophagus. This procedure was introduced in the 1970s and remains safe, well tolerated and efficacious with a relative low risk of serious complications. Another benefit to this procedure is the ability to adjust the band enhancing its weight loss effect without compromising safety [4].

### Roux-en-Y Gastric Bypass

The Roux-en-Y gastric bypass (RYGB) is one of the most common bariatric procedures and has the greatest weight loss effect [5]. During the procedure, a small gastric pouch is created, draining into the jejunum (alimentary limb) causing nutrients to bypass the pylorus and duodenum. The bile and pancreatic juices drain into the duodenum and jejunum as normal (biliopancreatic limb) but are only mixed with food after the anastomosis of the alimentary and biliopancreatic limbs to create the common limb. The length of the common limb is an important factor for the development of serious complications [5]. In standard RYGB, the Roux limb is usually 0.75–1.5 m long with a common limb of ∼3 m which is usually adequate for absorption of nutrients. A modified version of the RYGB is called ‘distal bypass’ technique, reducing the length of the common limb to ∼75 cm, carrying though a higher risk for complications [5].

### Sleeve Gastrectomy

The sleeve gastrectomy (SG) involves creating a long, thin longitudinal gastric pouch or sleeve. This reduces the volume of the stomach by approximately 80% but leaves the pylorus intact. SG was initially performed as a precursor to a larger procedure but has been increasingly used alone due to its efficacy and safety. SG is now one of the most commonly performed bariatric surgery procedures with impressive weight loss effect and relative low rate of complications [6].

## Potential Hormone Mechanisms of Bariatric Surgery

**Fig. 1:**
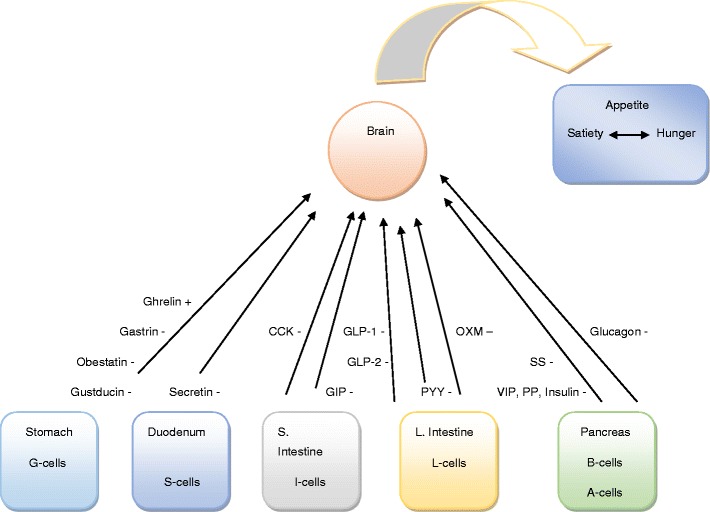
Hormone interactions (feedback mechanisms) between the brain and organs of the gastrointestinal tract

The metabolic effects of bariatric surgery have been attributed by many to changes in the secretion of gastroenteropancreatic peptides although additional mechanisms have been proposed (Fig. 1). In this review article, we will focus on the weight loss-independent effects of bariatric surgery mainly involving changes in postprandial gut hormone secretions (Table 2) [3].

**Table 2:** Gut hormones and their actions

Hormone	Organ/cell	Mechanism of action
Gastrin	Stomach/G cells	Increases HCl production
		Promotes satiety
Ghrelin	Stomach/G cells	Increases appetite
		Enhances gastric emptying GI motility and GH secretion
GLP-1	Ileum/L cells	Causes the incretin effect
		Increases insulin sensitivity and production. Delays gastric emptying. Enhances satiety.
GLP-2	Ileum/L cells	Causes gut hypertrophy. Alters GI motility
PYY	Colon/L cells	Delays gastric emptying. Promotes satiety
OXM	Ileum/L cells	Promotes satiety, increases energy expenditure
Secretin	Duodenum/S cells	Reduces gastric and duodenal motility. Enhances insulin release
VIP	Enteric and parasympathetic nerves	Promotes hormone secretion by the brain, gut and pancreas Increases the secretion of water and electrolytes. Reduces HCl secretion
PP	Pancreas- PP cells	Promotes satiety
		Exocrine and endocrine secretion regulator
Insulin	Pancreas/B cells	Regulates metabolism of carbs, fat and protein. Promotes absorption glucose from the blood
Obestatin	Stomach/epithelial cells	Promotes satiety
Gustducin	Stomach/specialized lining cells	Enhances GLP-1 secretion
SS	Pancreas/D cells	Reduces gastrin, GLP-1, CCK, GIP and secretin
Glucagon	Pancreas/A cells	Promotes glucogenolysis and gluconeogenesis
FGF19	Ileum	Regulation of glucose and lipid metabolism. Increases energy expenditure

## Bariatric Surgery and Gut Hormones

### Gastrin

Gastrin is a peptide hormone that stimulates the secretion of gastric acid (HCl) by the parietal cells of the stomach and aids in gastric motility. It is released by G cells in the pyloric antrum of the stomach, duodenum and the pancreas. Gastrin binds to cholecystokinin B receptors to stimulate the release of histamines in enterochromaffin-like cells, and it induces the insertion of K+/H+ ATPase pumps into the apical membrane of parietal cells (which in turn increases H+ release into the stomach cavity). Its release is stimulated by peptides in the lumen of the stomach and gastrin reduces appetite. Rather than being a single molecular entity, gastrin is actually a family of multiple peptides of varying lengths with varying degrees of biological activity. As one would expect, following RYGB, there is some evidence suggesting that postprandial gastrin levels fall after RYGB both in the first 2 weeks postoperatively [7•] and over the first year [8]. Recent evidence suggests that the remnant stomach is subject to multiple histological changes following RYGB. One study demonstrated increased cell proliferation rate in the epithelium of the excluded gastric antrum coupled with a reduction in the number of G cells [9]. It has been suggested that excessive gastric acid production maybe involved in the pathogenesis of abnormal histological findings in the stomach after RYGB [10]. Grong et al. used a protein-rich mixed meal on a cohort of 20 female patients previously operated with RYGB or SG versus 13 female matched controls and were able to demonstrate diminished gastrin levels in the RYGB but no statistically significant changes between the SG group and controls [11]. A study of 24 patients following GB demonstrated no change in fasting gastrin concentrations 6–12 months after surgery [12]. Others have showed that SG may be associated with increased gastrin levels in both human and rodents [13, 14]. The role of gastrin secretion as either a cause or a consequence of altered gastric histology remains unclear.

### Ghrelin

Ghrelin is a peptide hormone produced by ghrelinergic cells in the gastrointestinal tract and functions as a neuropeptide in the central nervous system [15•]. Besides regulating appetite, ghrelin also plays a significant role in regulating the distribution and rate of use of energy [16]. Ghrelin is most active in its acylated form, with an octanoyl group attached to its third amino acid residue, which occurs due to the action of ghrelin-*O*-acyltransferase (GOAT). As a consequence, it activates the growth hormone secretagogue receptor (GHSR), which is predominantly found in the hypothalamus and pituitary glands [17]. Ghrelin levels rise with prolonged fasting and drop after ingestion of food, hence the poor long-term efficacy of diet for the management of obesity [18•]. The effect of bariatric procedures appears to have variable effects on ghrelin secretion, possibly due to the altered passage of ingested nutrients through the gastric fundus where the ghrelin-producing cells are predominantly located and additionally due to small sample size of the existing studies. Fruhbeck and co-workers studied 24 obese men following adjustable GB (*n* = 8), RYGB (*n* = 8) or lifestyle modifications [19, 20]. Six months after surgery, patients with GB and lifestyle group had similar ghrelin levels, whereas patients with RYGB had a significant decrease. Cummings et al. noted that gastric bypass (*n* = 5) was associated with a reduction in 24-h ghrelin area under the curve when compared to five healthy obese matched controls and ten controls of normal weight [19]. Dirksen et al. followed 33 patients for 12 months after RYGB and observed that greater weight loss was associated with a higher degree of ghrelin suppression postsurgically [21•]. Others, however, have demonstrated increased ghrelin levels in humans or rodents following RYGB [22, 23]. On the other hand, SG may be associated with reduced ghrelin levels likely due to the removal of that part of stomach where ghrelin-secreting cell concentration is higher [24].

### Cholecystokinin

Cholecystokinin (CCK) is a peptide hormone of the gastrointestinal system, synthesized and secreted by I cells in the duodenal mucosa being responsible for stimulating the digestion of fat and protein. Its presence causes the release of digestive enzymes and bile from the pancreas and gallbladder, respectively, slowing gastric emptying and also acting as a hunger suppressant. Several studies have shown an increase in CCK levels postprandially after RYGB in response to a mixed meal [7•, 21•]. CCK is primarily released in the presence of amino acids and fatty acids within the duodenum, which is excluded from contact with nutrients following RYGB in contrast with what studies have shown. Other factors of CCK release such as parasympathetic impulses and intraluminal releasing factors may be responsible for the increased levels of CCK following bariatric surgery [25]. The effect of bariatric surgery on CCK homeostasis still remains unclear. Few studies have examined the changes in CCK which occur following other bariatric procedures. Mans et al. showed that SG resulted in enhanced CCK levels and increased satiety in 8 morbidly obese patients versus 16 matched controls [26]. Peterli et al. [27•] studied patients for a year after RYGB or SG. SG was associated with a much larger CCK increase compared to the RYGB group. The effect of SG in CCK secretion was evident 1 week postoperatively and gained in magnitude over the first year [27•]. To date, there are no studies looking at the effect of GB on CCK concentration.

### Glucose-Dependent Insulinotropic Polypeptide

Glucose-dependent insulinotropic polypeptide (GIP) is derived from a 153-amino acid proprotein encoded by the GIP gene and circulates as a biologically active 42-amino acid peptide. It is synthesized by K cells, which are found in the mucosa of the duodenum and the jejunum of the gastrointestinal tract. Like glucagon-like peptide-1 (GLP-1), GIP is associated with an insulinogenic effect following ingestion of oral glucose, known as the incretin effect [28]. The role of GIP in the development of diabetes and obesity is unclear, but hyperglycaemia may act to directly down-regulate GIP receptors in pancreatic b-cells leading to a defect in late-stage insulin release. GIP is also thought to have significant effects on fatty acid metabolism through stimulation of lipoprotein lipase activity in adipocytes [29]. RYGB is found in some studies to cause a reduction in postprandial GIP secretion due to the restriction of nutrient passage through the duodenum and jejunum, and this effect may be enhanced in patients with type 2 diabetes mellitus (T2DM) [30–32]. Bunt et al. assessed the effects of a mixed-meal test following GB and RYGB and found statistically significant lower GIP levels in the RYGB group [33]. The RYGB group had increased GIP levels postsurgically whereas there were no observed changes in the GB group. Similar findings have been reported by others after GB [34]. The effects of SG on GIP regulation have not been studied sufficiently.

### Glucagon-Like Peptide 1

GLP-1 is a 30-amino acid-long peptide hormone deriving from the tissue-specific posttranslational processing of the proglucagon gene. It is produced and secreted by intestinal enteroendocrine L-cells and certain neurones within the nucleus of the solitary tract in the brainstem upon food consumption. Alongside GIP, GLP-1 is the only known incretin describing its ability to decrease blood sugar levels in a glucose-dependent manner by enhancing the secretion of insulin. Beside the insulinotropic effects, GLP-1 has been associated with numerous regulatory and protective effects. Despite the lack of evidence indicating increased GLP-1 concentrations following bariatric surgery [35], postprandial GLP-1 levels are increased following GB, SG and RYGB [36, 37]. It is not fully understood why there is increase in GLP-1 postsurgery, but it is believed that it may be associated to the passage of more intact nutrients to the ileum through anatomical changes or increased intestinal transit [25, 38]. Others suggest that bypassing the upper small intestine may be responsible for the beneficial effect of bariatric surgery [39, 40]. Supporting evidence for the importance of the GLP-1 system comes from acute human experiments in which GLP-1 antagonists are administered to humans after an RYGB and appear to reduce the enhanced insulin secretion that occurs after the procedure [41••, 42•]. However, in genetic loss-of-function experiments, where mice lack the only identified receptor for GLP-1, both vertical sleeve gastrectomy (VSG) and RYGB have identical effects on both weight loss and glucose improvements compared with the effects in wild-type mice, implying that activation of the GLP-1 receptor does not contribute to the benefits of VSG and RYGB [43, 44•]. GLP-1 agonists or mimetics such as liraglutide, exenatide or lixisenatide have been approved for pharmacological use in the treatment of diabetes and obesity [45, 46]. GLP-1 is also thought to have centrally mediated effects upon appetite by interacting with vagal afferent nerve fibres. In rodents, GLP-1 administration appears to activate neurones in the arcuate nucleus and paraventricular nucleus to promote satiety [47–50]. Performing a vagotomy with bariatric surgery attenuates these effects [48]. There is also some evidence to support an increase in energy expenditure in rodents [50], but this has not been replicated in humans. Dirksen et al. showed that levels of GLP-1 were higher in patients who lost more weight, compared to those with poor weight loss [21•]. In another study, SG was still effective in GLP-1 receptor knockout mice, suggesting that alternative pathways must also be involved [51]. Mokadem et al. revealed that RYGB had beneficial metabolic effects in two GLP-1 KO mouse models, similar to RYGB-treated control mice [52•]. Although GLP-1-induced incretin effect might be in part responsible for the improved glucose tolerance after bariatric surgery, increased insulin secretion might be expected to produce weight gain, rather than weight loss. The weight loss-induced effect of GLP-1 following bariatric surgery remains poorly understood.

### Glucagon-Like Peptide 2

Glucagon-like peptide 2 (GLP-2) is a 33-amino acid peptide created by specific posttranslational proteolytic cleavage of proglucagon in a process that also liberates the related GLP-1. GLP-2 is produced by the intestinal endocrine L cell and by various neurones in the central nervous system. Intestinal GLP-2 is co-secreted along with GLP-1 upon nutrient ingestion. GLP-2 appears to have a role in stimulating gut hypertrophy by ileal cell hyperplasia and reducing apoptosis and has been used therapeutically in patients with short gut syndrome [53, 54]. There are various studies assessing the effects of RYGB on GLP-2 regulation. Taqi et al., in an experimental study, demonstrated a significant increase in the GLP-2 levels after gastric bypass in rats [55]. LeRoux et al., in a human prospective study, demonstrated a significant increase in the postprandial levels of GLP-2 after gastric bypass, with a secretion peak observed 6 months after the procedure [56]. Cazzo et al., in a human prospective study, observed a significant increase in the GLP-2 levels 12 months after surgery and demonstrated that this increase was significantly correlated with aspects of satiety regulation [57]. Comparing individuals who underwent gastric bypass and SG, Romero et al. observed in a prospective study that both procedures led to a significant increase in the postprandial levels of GLP-2 6 weeks after surgery, without significant difference between the two evaluated procedures [58]. Cummings et al., in an experimental study, demonstrated a significant increase in the GLP-2 levels in rats after the SG [59•]. Evidence suggests that GLP-2 postoperatively increases, and this change may be potentially related to weight loss stabilization, late reduction of diarrhoea and malabsorption, partial compensation of harms to bone mineral metabolism, minimization of the consequences of bacterial overgrowth and regulation of specific aspects of satiety regulation.

### Pancreatic Peptide YY

Peptide YY (PYY) also known as pancreatic peptide YY3–36 is a peptide that in humans is encoded by the PYY gene. PYY is a short (36-amino acid) peptide released by L enteroendocrine cells in the distal small intestine and colon in response to feeding. Following cleavage in the circulation by the enzyme dipeptidyl-peptidase-IV (DPP-IV), PYY1–36 is converted to PYY3–36, suspected to promote satiety through its agonism of the Y2 receptor [60, 61]. PYY3–36 has many effects, including delaying gastric emptying, reducing postprandial insulin production and altering colonic motility, but its main role appears to involve the central regulation of appetite [60–63]. Le Roux et al. were able to demonstrate that obese individuals had reduced postprandial PYY3–36 levels and individuals infused with PYY3–36 demonstrated reduced food intake [64•]. Levels of PYY3–36 appear to increase postprandially following bariatric surgery [65••]. PYY3–36 levels increase postprandially regardless of bariatric procedure [37, 65••, 66•]. In rodent KO models, PYY appears to have an important role to weight loss following bypass surgery [25, 67]. Further research and innovative approaches are required to better understand PYY (3–36) physiology, its role in obesity and bariatric surgery and therapeutic potential.

### Oxyntomodulin

Oxyntomodulin originates from the proglucagon gene by alternative posttranslational processing pathways. It is a naturally occurring 37-amino acid peptide structurally similar to glucagon with an additional C-terminal octapeptide. Oxyntomodulin is produced by the L cells in the colon and has been found to suppress appetite. The mechanism of action of oxyntomodulin is not well understood. It is known to bind both the GLP-1 receptor and the glucagon receptor, but it is not known whether the effects of the hormone are mediated through these receptors or through an unidentified receptor. Oxyntomodulin regulation following bariatric surgery is poorly understood, and very few studies have looked at the effects of surgery on oxyntomodulin. In one study, ten obese women with T2DM who had RYGB were matched with ten women who achieved 10-kg weight loss through diet. The group who underwent RYGB had increased oxyntomodulin levels following a mixed-meal test that were correlated to circulated GLP-1 and PYY levels [68•]. These findings have been replicated by others [69••]. Exogenous administration of oxyntomodulin in mice leads to body weight loss, increased energy expenditure and amplified glucose-induced insulin secretion [70•, 71]. No specific receptor for oxyntomodulin has yet been identified, and because oxyntomodulin lacks the ability to increase insulin secretion in GLP-1R−/− mice, this effect of oxyntomodulin can likely be attributed to its action on GLP-1R. Subcutaneous oxyntomodulin infusions reduce body weight in rodents [70•] and humans by increasing energy expenditure and reducing food intake [72••, 73].

### Secretin

Secretin is a 27-amino acid peptide hormone which is produced by S cells in the duodenal mucosa in response to a low intraluminal pH, inhibiting the secretion of gastric acid from the parietal cells of the stomach and stimulating the production of bicarbonate from the centroacinar cells and intercalated ducts of the pancreas. It also stimulates bile production by the liver; the bile emulsifies dietary fats in the duodenum so that pancreatic lipase can act upon them. In humans, the secretin peptide is encoded by the SCT gene [74]. Rhee et al. studied the impact of Roux-en-Y gastric bypass (RYGB) on the density and hormonal gene expression of small-intestinal enteroendocrine cells in obese patients with type 2 diabetes. Twelve patients with diabetes and 11 age- and BMI-matched controls underwent RYGB followed by enteroscopy ~10 months later. Mucosal biopsies taken during surgery and enteroscopy were immunohistochemically stained for secretin and secretin mRNA was reduced after RYGB [75•]. In another study, 18 patients without T2DM had perianastomotic jejunal biopsies at baseline and using endoscopy 12 months postoperatively. RYGB had no impact on villi length or density of secretin [76]. Changes in secretin homeostasis have not been studied following GB or SG.

### Vasoactive Intestinal Polypeptide

Vasoactive intestinal polypeptide (VIP) is a neuropeptide of 28-amino acid residues that belongs to a glucagon/secretin superfamily, the ligand of class II G protein-coupled receptors, and is released by the enteric-neural system and parasympathetic efferent nerve fibres. It acts to increase the secretion of water and electrolytes into the pancreatic juices and the gut itself. VIP stimulates contractility in the heart, causes vasodilation, increases glycogenolysis, lowers arterial blood pressure and relaxes the smooth muscle of trachea, stomach and gall bladder. In humans, VIP is encoded by the VIP gene [77]. The effects of bariatric surgery on VIP regulation remain elusive.

### Pancreatic Polypeptide

Pancreatic polypeptide is a polypeptide secreted by pancreatic polypeptide (PP) cells in the endocrine pancreas predominantly in the head of the pancreas. It consists of 36 amino acids. The function of PP is to self-regulate pancreatic secretion activities (endocrine and exocrine); it also has effects on hepatic glycogen levels and gastrointestinal secretions. Its secretion in humans is increased after a protein meal, fasting, exercise and acute hypoglycaemia and is decreased by somatostatin and intravenous glucose [78]. Dixon et al. examined 17 postoperative individuals who had already achieved a mean of 28% GB-induced weight loss (range, 10–38%) whilst taking part in a cross-sectional study and 16 obese individuals prior to GB from a prospective study and assessed plasma PP and PYY meal responses. They concluded that PP responses appeared unchanged by weight loss status but a reduced PP meal response may predict higher weight loss [79].

### Insulin

Insulin is a 51-amino acid peptide hormone produced by beta cells of the pancreatic islets. It regulates the metabolism of carbohydrates, fats and protein by promoting the absorption of, especially, glucose from the blood into fat, liver and skeletal muscle cells. Accumulating evidence suggests that β-cell function can be improved early after RYGB and SG. In glucose-tolerant individuals, the insulin response to a mixed meal is decreased after weight loss resulting from GB, SG or RYGB, Specifically, the profile of the insulin curve shifts to a more rapid response and a steeper fall after SG and RYGB, whereas the insulin curve after GB is characterized by a parallel downshift of the insulin concentration [7•, 80••, 81•, 82•]. The different profiles are probably caused by the different rates of glucose absorption and especially the different GLP-1 response profiles [7•, 80••]. In cases of type 2 diabetes, only minor changes in insulin profile have been reported after GB, whereas after SG or RYGB, an improved initial insulin response has been described that is reminiscent of that in glucose-tolerant obese people [83•]. B-cell glucose sensitivity increases a few days after SG or RYGB, thus improving the dynamic responsiveness during a meal, whereas the responsiveness to intravenous glucose stimulation is unchanged during the first few months after surgery [82•, 83•]. Additionally, insulin sensitivity also improves after bariatric surgery. Regardless of the type of operation, hepatic insulin sensitivity is improved within days after the procedure, presumably due to caloric restriction. Following major weight loss occurring after several months postsurgically, insulin sensitivity is also improved in individual cases [82•, 83•]. Weight loss effect therefore, appears to have a catalytic role regarding the magnitude of improvement in skeletal muscle insulin sensitivity. Of patients following RYGB, SG and GB, respectively, 1, 4 and 31% have unchanged glucose tolerance [84]. Bariatric surgery also reduces the likelihood of progression to T2DM compared to matched controls [85••].

### Obestatin

Obestatin is a hormone that is produced in specialized epithelial cells of the stomach and small intestine of several mammals including humans. Obestatin was originally identified as an anorectic peptide, but its effect on food intake remains controversial [86]. Obestatin is encoded by the same gene that encodes ghrelin, a peptide hormone. Ghrelin is cleaved to produce proghrelin which is cleaved to produce a 28-amino acid ghrelin (unacylated) and C-ghrelin(acylated). Obestatin is presumed to be cleaved from C-ghrelin [87]. Zhou et al. were able to demonstrate increased obestatin and ghrelin/obestatin ratio in mice treated with RYGB whereas obestatin was decreased in the SG group [25]. In a human study, Siejka et al. were unable to demonstrate significant changes in obestatin levels post-SG [88]. In contrast to ghrelin, which acts as an appetite stimulant, treatment of rodents with obestatin suppresses food intake, inhibits jejunal contractions and decreases body weight [89]. Only a few studies on obestatin levels in human obesity have been published. Plasma obestatin levels are significantly lower in obese subjects, as compared to lean controls, indicating a role for obestatin in long-term body weight regulation, and decreased obestatin levels were reported in morbidly obese subjects referred to bariatric surgery.

### Gustducin

Whilst gustducin was known to be expressed in some taste receptor cells (TRCs), studies with rats showed that gustducin was also present in a limited subset of cells lining the stomach and intestine. These cells appear to share several features of TRCs [90]. Recent rodent studies have found functional intestinal nutrient sensing through α-gustducin in the gut in relation to GLP-1 secretion after RYGB [52•]. Under normal circumstances, T1r3 sweet taste receptors on the L cells are coupled to the G protein α-subunit α-gustducin. Activation of this pathway stimulates GLP-1 secretion. As a result, α-gustducin−/− knockout mice, similar to GLP-1R KO, are considered a functional knockout of GLP-1 signalling [52•]. In another rodent study, the role of α-gustducin in the RYGB-induced improvement of glucose homeostasis could not be clearly assessed by Steensels et al. as sham-operated *α-gust KO* mice displayed better glucose profiles and tended to display lower insulin levels compared to sham-operated wild-type (WT) mice [91]. These results indicate that *α-gust KO* mice were partially protected from the diabetogenic properties of a western style diet. Avau et al. previously showed that high-fat diet-induced obese *α-gust* KO mice have an increased heat production compared to WT mice, as a result of an increased brown adipose tissue thermogenic activity [92]. Finally, α-gustducin stimulates the expression of sodium glucose transporter and Glut2 receptor, and activation of α-gustducin may be correlated to the hyperabsorption observed during an oral glucose tolerance test following bariatric surgery [93].

### Somatostatin

Somatostatin is a peptide hormone that regulates the endocrine system and affects neurotransmission and cell proliferation via interaction with G protein-coupled somatostatin receptors and inhibition of the release of numerous secondary hormones. Somatostatin inhibits insulin and glucagon secretion. Somatostatin is a peptide hormone produced by delta (or D) cells in the pancreas, stomach and duodenum. The pre-pro-hormone can be cleaved at two different locations giving two forms of 14 and 28 amino acids in length, which both have biological activity. In the gastrointestinal tract, it reduces the secretion of gastrin, secretin, CCK, GIP and GLP-1. In the pituitary, it reduces the secretion of growth hormone, thyroid-stimulating hormone and prolactin. In the pancreas, it reduces the production and secretion of insulin and glucagon and inhibits exocrine secretion. A somatostatin analogue with high biding affinity to somatostatin receptor 2 and lower to 3 and 5 (sstr 2,3,5), octreotide, has caused reduced adiposity in high-fat-fed rats [94]. Somatostatin infusion in a study of obese women inhibited release of PYY [95]. Despite the previous, the role of somatostatin following bariatric surgery remains poorly characterized.

### Glucagon

Glucagon is a 29-amino acid peptide hormone produced in the alpha cells of the pancreatic islets of Langerhans, which are located in the endocrine portion of the pancreas. Its production is suppressed/regulated by insulin from the adjacent beta cells. Glucagon is also released during the fasting state and acts to increase blood sugar levels by promoting glycogenolysis and gluconeogenesis. Very few studies have assessed the effect of bariatric surgery upon circulating glucagon concentrations. Farey et al. demonstrated a significant reduction in glucagon levels 3 months post-SG [96]. Korner et al. assessed women after GB and RYGB and in a group of overweight control patients (*n* = 36 in total) for glucagon concentrations. The investigators found no difference in glucagon levels between groups following a mixed-meal test, except for 180 min after the test was commenced where patients who had the RYGB compared to the control group had significantly lower glucagon levels [97]. Umeda et al. assessed glucagon concentrations at baseline and 3 months after RYGB. According to their observations, fasting glucagon concentrations increased and postprandial glucagon levels decreased following surgery in response to a liquid meal [98].

### Fibroblast Growth Factors (FGF19,21)

Fibroblast growth factors (FGFs) constitute a family of proteins comprising at least 22 members involved in the regulation of cell growth and differentiation, development, angiogenesis, wound repair and metabolism [99]. Most FGFs are secreted heparin-binding proteins and function as autocrine or paracrine factors, whilst FGF19, FGF21 and FGF23 exhibit common unique structural properties which confer them the ability to elicit endocrine actions functioning as hormones. FGF19 is an ileum-derived enterokine that controls bile acid and nutrient metabolism. FGF19 has been reported to play a role in the regulation of glucose and lipid metabolism, as well as in energy expenditure and body adiposity [99]. FGF21 is produced mainly in the liver and promotes fatty acid oxidation, improves insulin sensitivity and increases energy expenditure [99]. FGF21 is paradoxically increased in obesity, suggesting that obesity is a FGF21-resistant state [99]. Gomez-Ambrosi et al. were able to demonstrate that FGF19 levels in obese patients increase after bariatric surgery-induced weight loss regardless of the surgical procedure used, but not after diet-induced weight loss [100]. They were also able to demonstrate that FGF21 concentrations in obese patients decrease after diet- and SG-induced weight loss, but not after weight loss following RYGB [100]. Martinez de la Escalera et al. investigated 39 obese women with T2DM who underwent various bariatric procedures and were able to demonstrate that changes in circulating FGF19 levels were surgery specific [101].

## Conclusions

Whether the metabolic benefits of bariatric surgery are secondary to weight loss or not remains controversial. It is nevertheless clear that there are widespread physiological responses to changes in GI tract morphology. Studies of peptide hormone concentrations after bariatric surgery have often found conflicting results, and these findings are further complicated by the fact that most of the observed hormonal and physiological responses are similar between all surgical interventions. Weight loss itself can cause changes in gut hormone secretion, regardless of the effects of bariatric surgery, making it difficult to evaluate the independent effects of surgery on weight loss postsurgically. The beneficial effects of bariatric surgery are still poorly understood, but are most likely to be multifactorial in aetiopathogenesis. An obvious candidate, however, is GLP-1. The increase in postprandial GLP-1 levels following bariatric surgery is obviously beneficial to the stimulation of postprandial insulin secretion. Interestingly, however, suppression of the GLP-1R does not diminish the beneficial effects of surgery, indicating that this is not the only mechanism for the observed metabolic phenomena of surgery. Changes in calorie restriction occurring postsurgery, coupled with hormonal adaptations, promote weight loss which, surprisingly, can be preserved in the long term. Yet, there are patients lacking sustainable weight loss despite initial good response to bariatric surgery (secondary poor responders). There are several hypotheses regarding this phenomenon, including cognitive traits related to eating behaviour and isolated cases of blunted GLP-1 and PYY secretion following bariatric surgery. In contrast, primary poor responders to bariatric surgery may be genetically predisposed to a limited weight loss as various genome-wide associated studies have shown. In such cases, weight loss % over time is a strong prognostic factor to overall achieved weight loss.

The success of bariatric surgery comes from the combined effect of physiological and molecular signalling changes in both the GI tract and other organs that result in sustained weight loss and improved glucose and insulin homeostasis.
